# Epithelial–Mesenchymal Transition (EMT)

**DOI:** 10.3390/ijms241411386

**Published:** 2023-07-13

**Authors:** Guidalberto Manfioletti, Monica Fedele

**Affiliations:** 1 Department of Life Sciences, University of Trieste, 34127 Trieste, Italy; 2 National Research Council (CNR), Institute of Experimental Endocrinology and Oncology (IEOS), 80145 Naples, Italy; mfedele@unina.it

Epithelial–mesenchymal transition (EMT) is a cellular process involved in many physiological and pathological conditions. However, in the last decade there has been a growing interest in this process, especially in the cancer field. It is now known that in epithelial-derived solid tumors, high tumor grades, lymph node metastases, and an advanced clinical stage are linked to EMT. The cellular and molecular processes with which these connections are created are gradually being defined and major discoveries have been published in recent years.

One of the hot topics of the last two years has been the association of EMT with the innate inflammatory response and, specifically, with neutrophil extracellular traps (NET). NETs are extracellular filamentous web-like structures of DNA and histones associated with cytotoxic enzymes and proteases, such as neutrophil elastase and metalloproteinase-9 (MMP-9), respectively. NETs have been shown to promote a prometastatic phenotype in human breast and gastric cancer cells by inducing EMT [1,2]. More recently, NETs have also been frequently found in colon cancer tissues, localizing to the invasive front and/or in the center of the tumor, where they activate the EMT, thus possibly contributing to the metastatic process [3]. Most interestingly, in triple-negative breast cancer models, chemotherapy induces IL1β release, which in turn causes neutrophil recruitment and NET formation, which counteract treatment efficacy by activating TGFβ-mediated EMT [4]. Another line of research, extensively developed in the last two years, concerns the N6-methyladenosine (m6A)-dependent regulation of EMT-related mRNAs. The addition of an m6A, one of the most prevalent internal modifications of eukaryotic mRNA, is a multi-step process performed by three classes of actors: the “writers”, represented by the m6A methyltransferases, which apply the modification; the “readers”, represented by m6A binding proteins, which recognize the modification; and the “erasers”, represented by the demethylases, which remove the modification [5]. Recent studies have indicated that m6A modifications of mRNA are increased in cancer cells during EMT and are functional to EMT induction and cancer progression. In colorectal cancer cells, the interaction of the long non-coding RNA MIR100HG and the heterogeneous nuclear ribonucleoprotein A2B1, an m6A reader, facilitates the m6A-dependent mRNA stabilization of TCF7L2, a downstream effector of Wnt signaling that is involved in EMT induction. This axis is particularly active in patients who have progressed after cetuximab treatment, suggesting a mechanism of resistance to this drug [6]. Two other m6A readers, IGFBP2 and YTHDF1, have been identified in head and neck squamous carcinoma (HNSCC) and breast cancer cells, respectively, as promoters of EMT and tumor progression [7,8]. IGF2BP2 recognizes and binds to the m6A site in the coding sequence region of Slug, thereby promoting its mRNA stability, suggesting it could act as a therapeutic target and prognostic biomarker for HNSCC patients with metastasis [7]. YTHDF1 binds to the m6A-modified mRNA of FOXM1, accelerating its translation process [8]. In breast cancer cells, FOXM1 promotes EMT by facilitating Slug expression [9], thereby promoting breast cancer metastasis [8]. 

Intratumor heterogeneity is recognized as a major cause of metastatic dissemination and colonization and of the conferral of resistance to existing therapies. This heterogeneity can be mainly attributed to the process of EMT that enables epithelial cells to enter a series of intermediate states, the so-called EMT continuum, and to its reverse mesenchymal–epithelial transition (MET), conferring a high level of phenotypic plasticity. Recent results pinpoint the relevance of epigenetic factors in controlling the epithelial–mesenchymal cellular plasticity. Two chromatin-modifying complexes with opposite functions, PRC2 (that catalyzes the di- and tri-methylation of K27 of histone H3, facilitating transcriptional repression) and KMT2D-COMPASS (that methylates histone H3 K4, facilitating gene expression), are involved in maintaining a stable epithelial state in EMT. Dysfunction of PRC2 or KMT2D-COMPASS unlocks two distinct mesenchymal trajectories resulting, for PRC2, in a quasi-mesenchymal state associated with highly metastatic capabilities and poor survival of patients with breast cancer [10]. Another epigenetic factor, the histone methyltransferase MLL3, a tumor suppressor frequently mutated in cancer, also plays a critical role in governing the hybrid EMT state. Indeed, MLL3 loss can exert a dual role in the EMT process by promoting EMT in epithelial cells and, conversely, MET in mesenchymal cells, leading to the acquisition of a hybrid EMT state with increasing metastatic colonization [11]. The crosstalk between epigenetics and splicing in EMT has been recently highlighted by the identification of KNF827 as a master regulator, able to drive EMT during cortical development and breast cancer metastasis. KNF827 can orchestrate a remodeling of the splicing landscape by recruiting HDAC1 on distinct genomic loci and modulating alternative splicing of key EMT regulators [12].

In this Special Issue, we have collected seventeen articles (seven literature reviews and ten original research articles), which address EMT and its role, regulation and targeting in cancer and other human diseases. Taiyab et al. contributed with an interesting original study about secondary cataracts, also known as posterior capsular opacification (PCO), in rat-lens epithelial cell (LEC) explants [13]. PCO arises when LECs are left behind in a capsular bag following surgery and are induced to undergo TGFβ-induced EMT [14]. The authors showed that the EMT process can be the result of crosstalk between the Smad3 and β-catenin pathways: the formation of a Smad3/MRTF-A complex binds to the β-catenin/CBP complex in the nucleus which then regulates EMT-related genes [13]. TGF-β-induced EMT was also the subject of three other research articles of this Special Issue [15,16,17]. Using the NMuMG cell line as a model of choice to study EMT, Schacke et al. demonstrated that TGFβ induces EMT concomitantly with an increased expression of Poly-adenosine diphosphate (ADP)-ribose (PAR), a polymer synthesized as a posttranslational modification by PARP-1/2, and is also associated with a PAR-belt disassembly while cells are changing in morphology to become mesenchymal. Then, they showed that the TGFβ-induced EMT in this model can be prevented, or partially reversed, by the poly (ADP-ribose) polymerase PARP-1/2 inhibitor Olaparib [15], a drug approved by the FDA to treat patients experiencing cancers with impaired homologous recombination [18]. Turini et al. investigated the consequences of TGFβ activation on the differentiation of human mesothelial cells exposed to chrysotile asbestos fibers, demonstrating that mesothelial cells undergo partial EMT after exposure to either TGFβ or chrysotile [16]. Xing et al. analyzed the role of Isoviolanthin, an extract from the medicinal herb and health food *Dendrobium officinale*, on the EMT of hepatocellular carcinoma cells, proving that it can counteract the TGFβ-induced activation of Smad- and PI3K-pathways, which regulate the TGFβ-induced EMT, migration and invasion [17]. 

On a different topic, Eva Delbrel et al. investigated whether the endoplasmic reticulum (ER) stress response and hypoxia are involved in idiopathic pulmonary fibrosis in which alveolar epithelial cells (AECs) acquire a mesenchymal phenotype through the EMT [19]; meanwhile, the article by Szynglarewicz et al. studied by IHC the expression of selected EMT markers in a cohort of ductal carcinoma in situ specimens, found on stereotactic breast biopsies, and correlated it with the risk of postoperative tumor invasion. They demonstrated that the preoperative expression of Snail and SPARC, but not N-cadherin, correlates with postoperative invasion [20]. 

A number of articles studied the effects of different compounds on EMT as an anti-cancer strategy. Among them, the study by Seba et al. analyzed the effects of two derivatives of chalcones, namely D14 and D15, on several osteosarcoma cell lines. The authors concluded that the chalcone drugs can inhibit the migration and invasion of osteosarcoma cells by regulating the expression of EMT-related genes, and that these effects are more pronounced in cells expressing wild-type p53 [21]. On this same issue, the article by Yang el al. describes the therapeutic potential of Bornyl cis-4-hydroxycinnamate in metastatic melanoma. The compound was tested in two melanoma cell lines with different metastatic capabilities and was found to reduce migration and invasion by inhibiting FAK/PI3K/Akt/mTOR and MAPK signaling pathways and EMT [22]. Romano et al. investigated the effect of Trefoil Factor 1 (TFF1) on the gastric cancer cell line AGS. They showed that the hypoxic conditions induced by gastric injury promote not only endogenous but also exogenous TFF1, which in turn can induce EMT, eventually triggered to repair the damage. Most interestingly, they also found that TFF1 itself can activate its promoter activity, thus resulting in a self-induction loop [23]. Finally, Jiang et al. revealed the anticancer effect of Sinomenine Hydrochloride against glioblastoma cells. Two cell lines were tested, and it was demonstrated that cell migration and invasion were reduced and the expression of EMT markers was reversed after the treatment. Mechanistically, they proved that Sinomenine Hydrochloride suppresses Matrix Metalloproteinase-2/-9 by inhibiting the NF-κB pathway [24]. 

Among the literature reviews, Tsubakihara and Mustakas beautifully discussed the central role played by TGFβ signaling in the induction of several EMT hallmark traits, including extracellular-matrix gene regulation, cell contact regulation, the regulation of the actin-based cytoskeleton, and the regulation of other growth factors and cytokines. The mechanisms of the TGFβ-induced EMT are presented by focusing especially on transcriptional factors involved in the EMT. Then, the authors discuss the significance of the EMT in cancer metastasis and the possibility to target the EMT in cancer treatment [25]. Kang et al. have discussed the role of altered glucose, lipids and amino-acid metabolic enzymes, their expression in tumors and the possible mechanism by which their alteration influences EMT induction in tumor cells. They emphasized the need for further studies on metabolic reprogramming to better understand tumor metabolism, with the aim to develop anti-tumor therapies to prevent tumor progression and improve anti-tumor therapeutic efficacy [26]. Another review dealing on the relevance of linking metabolic reprogramming, EMT and cancer, is the one provided by Revilla et al. This article presents interesting insights into the existence of a crosstalk between inflammatory mediators, such as the adipokines and metabolites of cholesterol, and EMT pathways in the context of thyroid carcinoma, placing particular emphasis on the relevance of cancer stem cells [27]. Along the same lines, Olea-Flores et al. addressed the connections between leptin signaling and EMT in breast cancer by presenting a survey of the most important contributions in this field. Leptin signaling can activate/repress major transcriptional factors that drive the gene expression reprogramming underlying epithelial loss and the expression of mesenchymal features related to loss of cell junctions and apico-basal polarity. The authors focused on recent advances that have established leptin as a risk factor for the development of breast cancer and its ability as an inducer of EMT in breast cancer cells [28]. Among the different mechanisms implicated in the regulation of the EMT, Gugnoni et al., reviewed lncRNA functions in this process. The authors described several examples of lncRNAs involved in promoting EMT, and other processes which are instead implicated in the inhibition of EMT, and their implications in cancer. Moreover, the authors discussed their prospective potential value as biomarkers and therapeutic targets in cancer [29]. The proneural–mesenchymal transition (PMT) may represent for glioblastoma, an extremely aggressive tumor of the central nervous system, the equivalent of EMT associated with other aggressive cancers. Fedele et al. framed the PMT in the high degree of phenotypic inter- and intra-tumor heterogeneity of GBM, which exists in different subtypes, each one characterized by further phenotypic variability in its stem-cell compartment. The authors discussed the significance of PMT in the acquisition of a multitherapy-resistance phenotype, to be taken in consideration for future perspectives in new anti-GBM therapeutic options [30].

Finally, Seccia et al. reviewed the role of EMT in hypertensive kidney disease. They stated that accumulating evidence implicates EMT in the development of renal fibrosis in several diseases, including hypertensive nephroangiosclerosis, and discussed studies that have investigated the role of EMT and its molecular mechanisms in hypertensive kidney disease [31].

In conclusion, this collection of articles shows that EMT is still on the crest of the wave as a potential target for the treatment of various human pathologies, especially cancer, to counteract its progression. The basic research reported in these articles is therefore fundamental for devising new therapeutic approaches.
